# Supplementary material for: Endocycle-related tubular cell hypertrophy and progenitor proliferation recover renal function after acute kidney injury
Source: Nat Commun. 2018 Apr 9;9:1344. doi: 10.1038/s41467-018-03753-4 (PMC5890293; doi:10.1038/s41467-018-03753-4)
Supplement: Supplementary file 1 — Supplementary Information [file 41467_2018_3753_MOESM1_ESM.pdf]

Endocycle-related tubular cell hypertrophy and progenitor proliferation recover renal function after acute kidney injury

Lazzeri et al.

Supplementary Information

Supplementary Figures

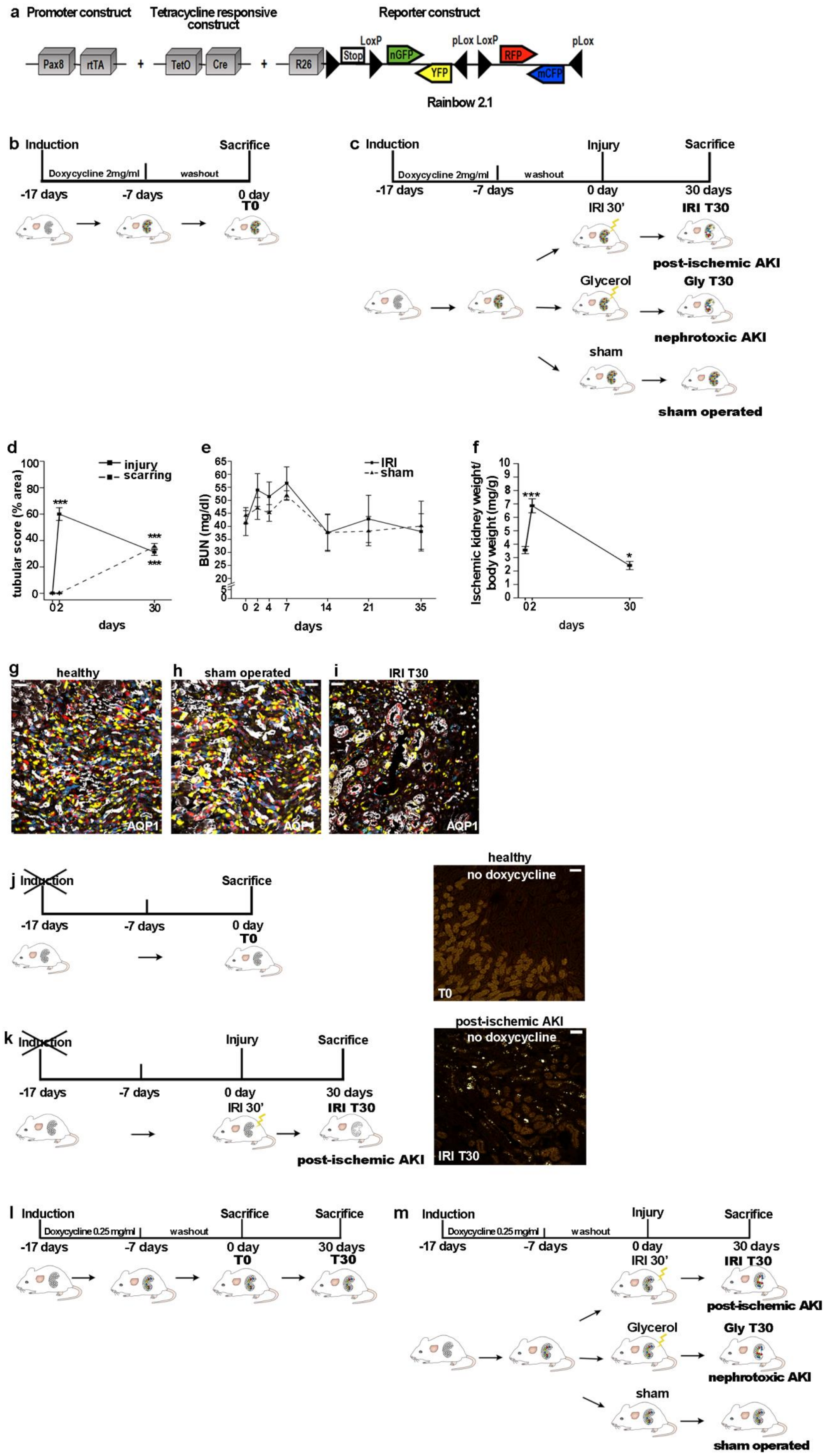
a Promoter construct
Tetracycline responsive construct
Reporter construct
Pax8
rtTA
TetO
Cre
R26
Stop
LoxP
nGFP
YFP
pLox LoxP
RFP
mCFP
pLox
Rainbow 2.1
b Induction
Sacrifice
Doxycycline 2mg/ml
washout
-17 days
-7 days
0 day
T0
c Induction
Injury
Sacrifice
Doxycycline 2mg/ml
washout
-17 days
-7 days
0 day
30 days
IRI 30'
IRI T30
post-ischemic AKI
Glycerol
Gly T30
nephrotoxic AKI
sham
sham operated
d
tubular score (% area)
injury
scarring
days
e
BUN (mg/dl)
IRI
sham
days
f
Ischemic kidney weight/ body weight (mg/g)
days
g healthy
h sham operated
i IRI T30
AQP1
j Induction
Sacrifice
-17 days
-7 days
0 day
T0
healthy
no doxycycline
k Induction
Injury
Sacrifice
-17 days
-7 days
0 day
30 days
IRI 30'
IRI T30
post-ischemic AKI
no doxycycline
l Induction
Sacrifice
Sacrifice
Doxycycline 0.25 mg/ml
washout
-17 days
-7 days
0 day
30 days
T0
T30
m Induction
Injury
Sacrifice
Doxycycline 0.25 mg/ml
washout
-17 days
-7 days
0 day
30 days
IRI 30'
IRI T30
post-ischemic AKI
Glycerol
Gly T30
nephrotoxic AKI
sham
sham operated

30

Supplementary Figure 1. Generation of Pax8/Confetti mice.

a) The inducible *Pax8.rtTA;TetO.Cre;R26.Confetti* (Pax8/Confetti) mouse is produced by crossing the *Pax8.rtTA* transgenic mice with *TetO.Cre* and *R26*-Confetti transgenic mice. The triple transgenic mouse constitutively expresses *rtTA* in Pax8+ cells but does not express the reporter proteins in any cell type while maintained on water not containing doxycycline. When included in the water, doxycycline binds to *rtTA*, allowing the transcription of Cre recombinase controlled by the *TetO* element. The Cre protein will specifically cut out or invert the floxed fluorochrome sequences and then turn on GFP, YFP, CFP or RFP expression. After doxycycline withdrawal, these Pax8+ cells will permanently express one out of four reporters, whereas any all that acquire Pax8 expression will not express the fluorescent reporters.

b, c) Experimental schemes.

d) Percentage of kidney area with tubular injury after IRI in Pax8/Confetti mice (n=5 mice per group). One-way ANOVA post-hoc Tukey; ***p<0.001 *vs.* day 0.

e) BUN levels in sham-operated mice and in ischemic Pax8/Confetti mice at different time points. Data are mean ± SEM from 5 mice in each group, one-way ANOVA post-hoc Tukey; NS at each time point IRI *vs.* sham-operated mice.

f) Kidney weight normalized to body weight after IRI in Pax8/Confetti mice (n=5 mice per group). One-way ANOVA post-hoc Tukey, ***p<0.001 and *p<0.01 *vs.* day 0.

g-i) Representative images of a kidney section of Pax8/Confetti mice after staining with anti-AQP1 antibody (white) in healthy (n=5) (g), in sham-operated mice (n=4) (h) and at day 30 after IRI (n=4) (IRI T30, i).

j, k) Experimental schemes and representative images of the absence of basal Cre recombinase activity in the kidney of Pax8/Confetti healthy mice (n=3) analysed at day 0 (T0) (j) and at day 30 after IRI (n=3, IRI T30) (k).

l, m) Experimental schemes.

Scale bars 40 µm.

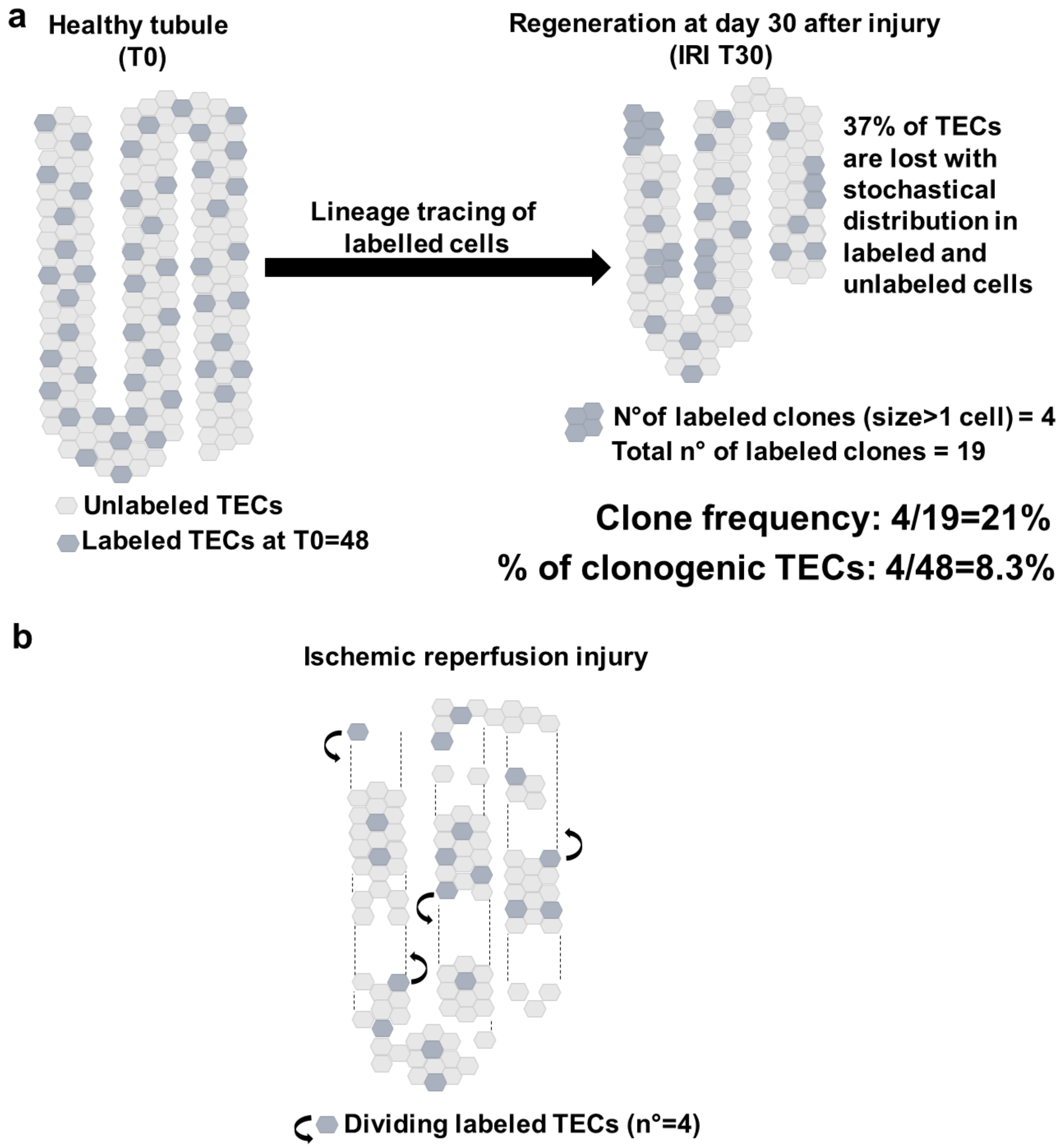

Supplementary Figure 2. Representative scheme of analysis performed in Pax8/Confetti mice

a) Representative scheme showing clone frequency and % of clonogenic TECs in Pax8 mice at day 30 after injury.

b) Representative scheme of TEC loss after ischemic reperfusion injury.

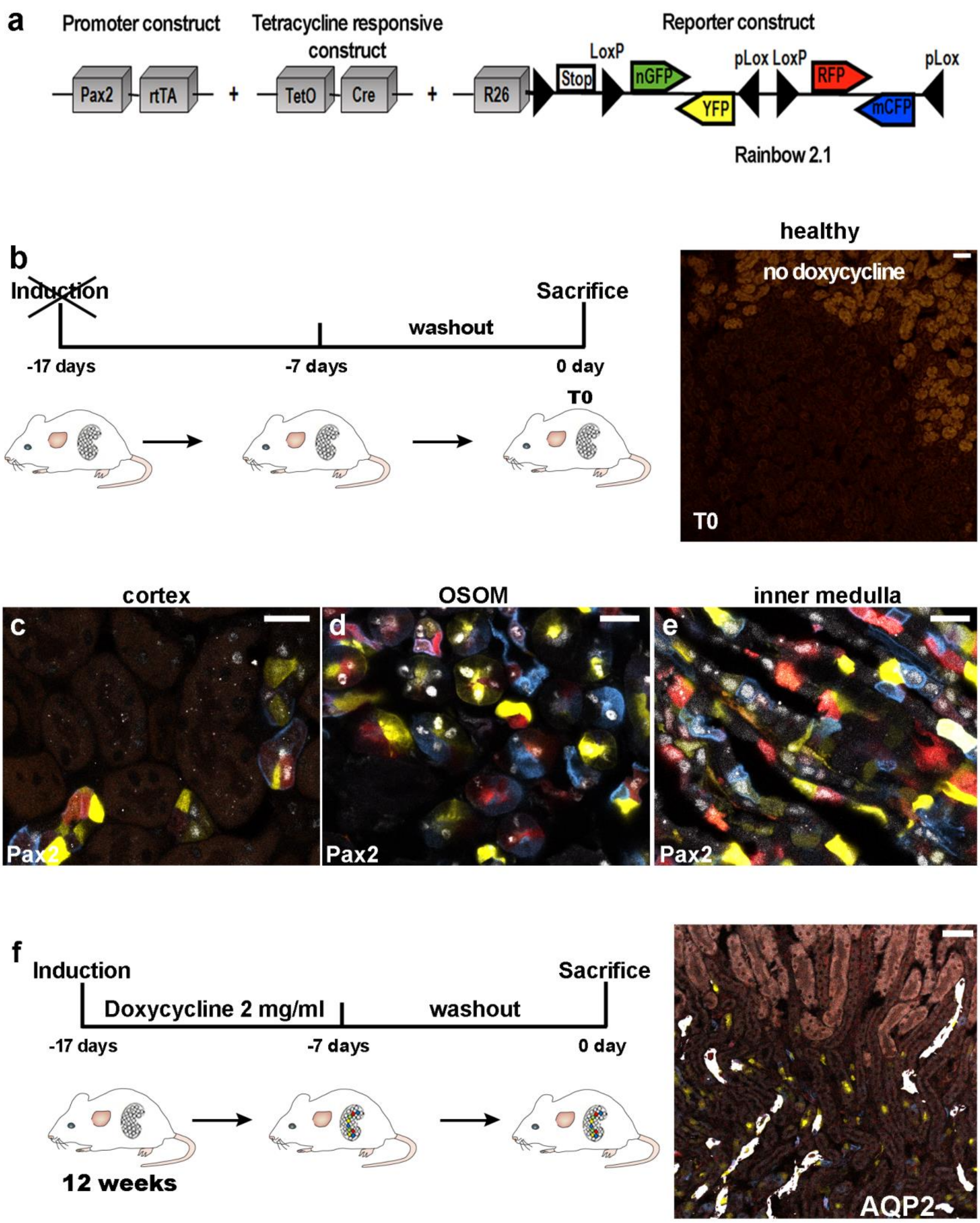

Supplementary Figure 3. Generation of Pax2/Confetti mice.

a) The inducible Pax2.rtTA;TetO.Cre;R26.Confetti (Pax2/Confetti) mouse is produced by crossing the Pax2.rtTA transgenic mice with TetO.Cre and R26-Confetti transgenic mice. The triple transgenic mouse constitutively expresses rtTA in Pax2+ cells but does not express the reporter proteins in any cell type while maintained on water not containing doxycycline. When included in the water, doxycycline binds to rtTA, allowing the transcription of Cre recombinase controlled by the TetO element. The Cre protein will

specifically cut out or invert the floxed fluorochrome sequences and then turn on GFP, YFP, CFP or RFP expression. After doxycycline withdrawal, these Pax2+ cells will permanently express one out of four reporters, whereas any all that acquire Pax2 expression will not express the fluorescent reporters.

b) Experimental scheme and representative image of the absence of basal Cre recombinase activity in the kidney of Pax2/Confetti healthy mice (n=3) analyzed at day 0 (T0). Scale bar 40 µm.

c-e) Representative images of a kidney section showing the staining of anti-Pax2 antibody (white) in the cortex (c), in OSOM (d) and in inner medulla (e) in healthy Pax2/Confetti mice (n=4). Scale bars 20 µm

f) Experimental scheme of Pax2/Confetti mice induced at 12 weeks of age and representative image of a kidney section after staining with AQP2 antibody (white) (n=3).

Scale bar 40 µm.

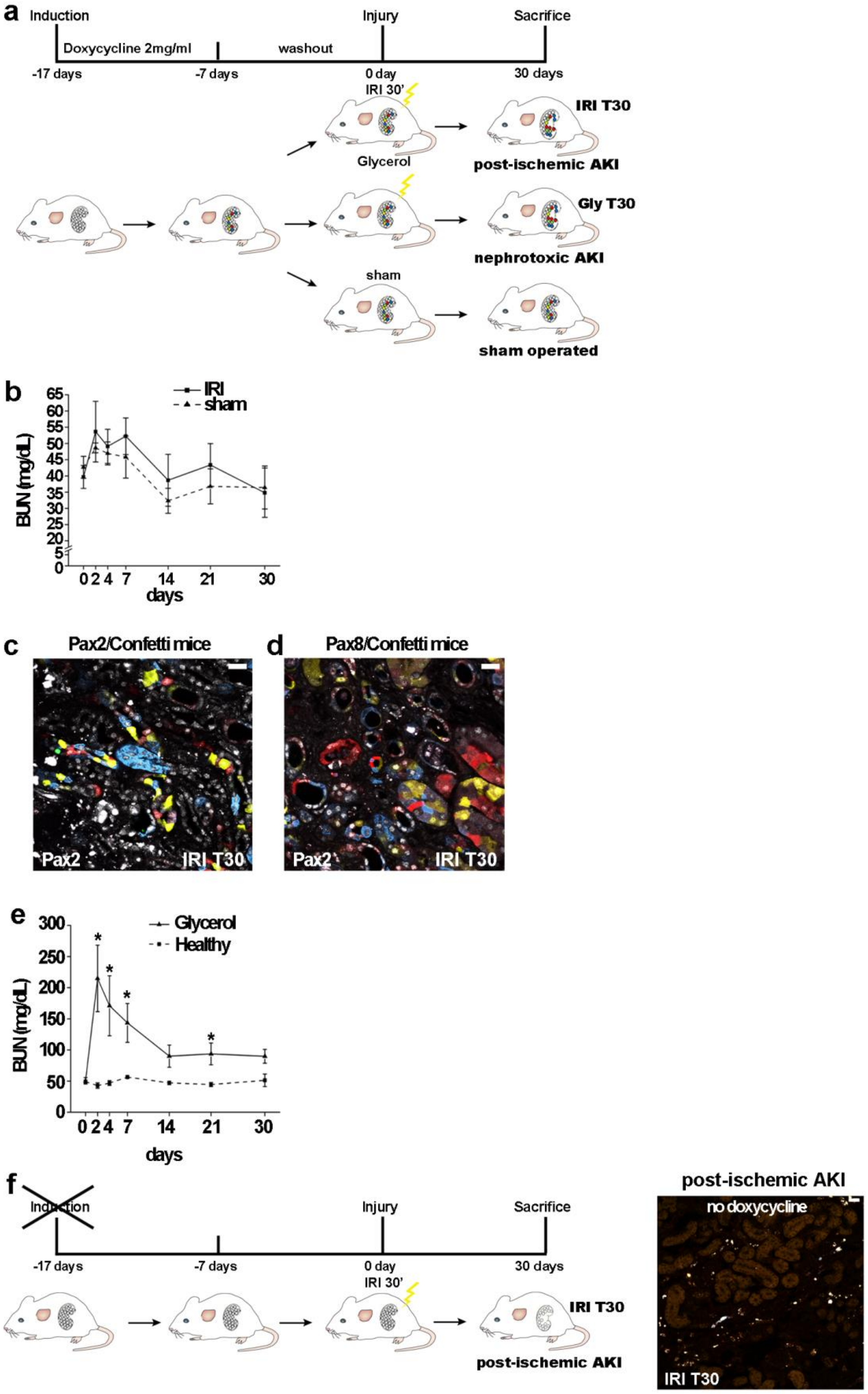

Supplementary Figure 4. Experimental scheme in Pax2/Confetti mice.

a) Experimental scheme

b) BUN levels in sham-operated mice and in ischemic Pax2/Confetti mice at different time points. Data are mean ± SEM from 5 sham-operated mice and 6 ischemic mice, one-way ANOVA post-hoc Tukey; NS at each time point IRI *vs.* sham-operated mice.

c, d) Representative images of a kidney section showing the staining of anti-Pax2 antibody (white) in OSOM of Pax2/Confetti mice (n=5) (c) and of Pax8/Confetti mice (n=4) (d) at day 30 after IRI. Scale bars 20 µm.

e) BUN measurement in healthy Pax2/Confetti mice (n=4) and after nephrotoxic AKI (n=6). One-way ANOVA post-hoc Tukey.

f) Experimental scheme and representative image of the absence of basal Cre recombinase activity in the kidney of Pax2/Confetti healthy mice (n=3) analyzed at day 30 after IRI (n=3, IRI T30).

Scale bar 40 µm.

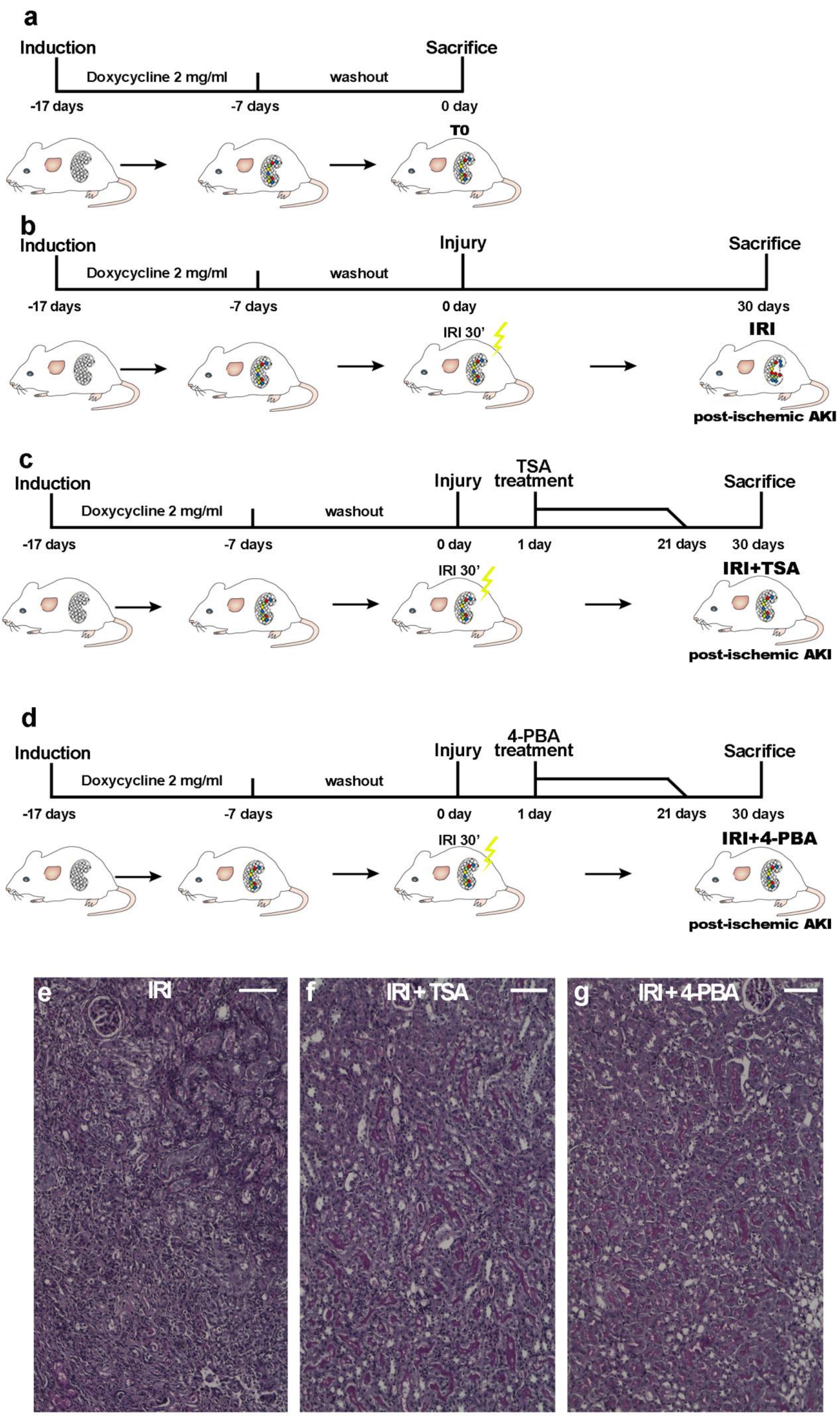

a
Induction
Sacrifice
Doxycycline 2 mg/ml
washout
-17 days
-7 days
0 day
T0
b
Induction
Injury
Sacrifice
Doxycycline 2 mg/ml
washout
-17 days
-7 days
0 day
30 days
IRI 30'
IRI
post-ischemic AKI
c
Induction
Injury
TSA treatment
Sacrifice
Doxycycline 2 mg/ml
washout
-17 days
-7 days
0 day
1 day
21 days
30 days
IRI 30'
IRI+TSA
post-ischemic AKI
d
Induction
Injury
4-PBA treatment
Sacrifice
Doxycycline 2 mg/ml
washout
-17 days
-7 days
0 day
1 day
21 days
30 days
IRI 30'
IRI+4-PBA
post-ischemic AKI
e
IRI
f
IRI + TSA
g
IRI + 4-PBA

Supplementary Figure 5. Experimental schemes of Pax2/Confetti mice treated with TSA and 4-PBA.

a-d) Experimental schemes.

e-g) Periodic Acid-Schiff (PAS)-stained kidney sections in Pax2/Confetti mice at day 30 after IRI (e) (n=6) and at day 30 after IRI + TSA (f) (n=4) or 4-PBA treatment (g) (n=6). Scale bars 100 µm.

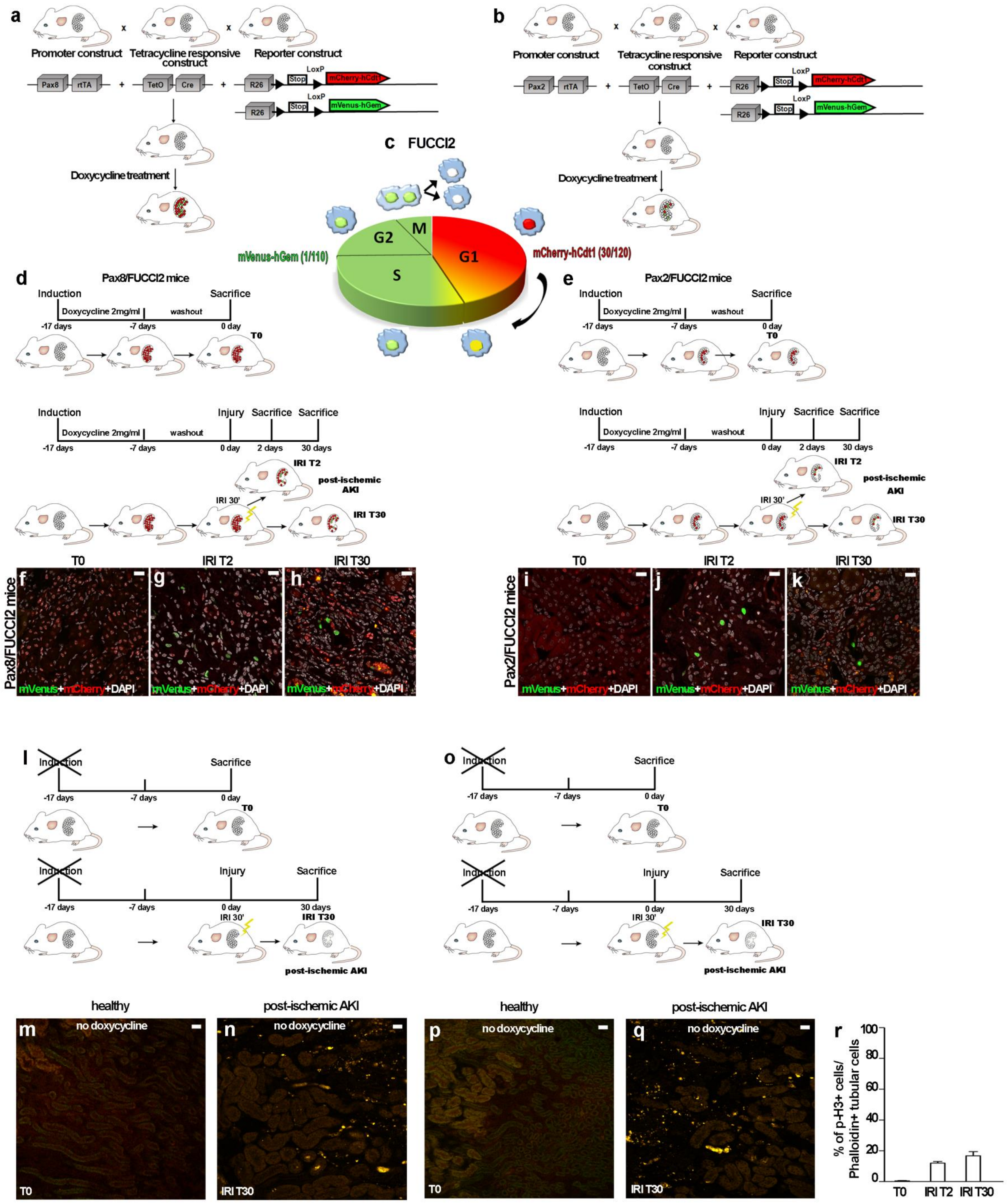

Supplementary Figure 6. Generation of inducible Pax8/FUCCI2 mice and inducible Pax2/FUCCI2 mice.

a) The inducible *Pax8.rtTA;TetO.Cre;R26.FUCCI2* (Pax8/FUCCI2) mouse is produced by crossing the *Pax8.rtTA* transgenic mice with *TetO.Cre* and *Rosa26*-FUCCI2 transgenic mice. The quadruple transgenic mouse constitutively expresses *rtTA* in Pax8+ cells but does not express the reporter proteins in any cell type

while maintained on water not containing doxycycline. When doxycycline is included in the water, Pax8+ cells express *rtTA* that binds the *TetO* element so that Cre recombinase expression is induced. The Cre protein will specifically cut out the floxed Stop cassette and then turn on mCherry-hCdt1(30/120) or mVenus-hGem(1/110) expression. Even after withdrawal of doxycycline from the water, these Pax8+ cells will permanently express the reporters, whereas any new Pax8+ cells that develop after doxycycline exposure will not express the fluorescent reporters.

b) The inducible *Pax2.rtTA;TetO.Cre;R26.FUCCI2* (Pax2/FUCCI2) mouse is produced by crossing the *Pax2.rtTA* transgenic mice with *TetO.Cre* and *Rosa26-FUCCI2* transgenic mice. The quadruple transgenic mouse constitutively expresses *rtTA* in Pax2+ cells but does not express the reporter proteins in any cell type while maintained on water not containing doxycycline. When doxycycline is included in the water, Pax2+ cells express *rtTA* that binds the *TetO* element so that Cre recombinase expression is induced. The Cre protein will specifically cut out the floxed Stop cassette and then turn on mCherry-hCdt1(30/120) or mVenus-hGem (1/110) expression. Even after withdrawal of doxycycline from the water, these Pax2+ cells will permanently express the reporters, whereas any new Pax2+ cells that develop after doxycycline exposure will not express the fluorescent reporters.

c) Schematic representation of cell-cycle phases labelled with mCherry-hCdt1(30/120) and mVenus-hGem (1/110) reporters. mCherry-hCdt1(30/120) is expressed in G1, whereas mVenus-hGem (1/110) is expressed in S/G2/M. Cells at the G1/S boundary express both mCherry-hCdt1(30/120) that mVenus-hGem (1/110). The FUCCI2 reporter has a small black phase when the cell goes out of mitosis. G1, gap 1 phase; S, synthesis; G2, gap 2 phase; M, mitosis.

d,e) Experimental schemes

f-h) mCherry+ cells (red) in G1 phase and mVenus+ cells (green) in S/G2/M phase of cell-cycle in Pax8/FUCCI2 mice at day 0 (n=4) (f), 2 (n=4) (g) and 30 after IRI (n=4) (h). DAPI counterstains nuclei (white). Scale bars 20 µm.

i-k) mCherry+ cells (red) in G1 phase and mVenus+ cells (green) in S/G2/M phase of cell-cycle in Pax2/FUCCI2 mice at day 0 (n=4) (i), 2 (n=4) (j) and 30 after IRI (n=4) (k). DAPI counterstains nuclei (white). Scale bars 20 µm.

l-n) Experimental scheme and representative images of the absence of basal Cre recombinase activity in the

kidney of Pax8/FUCCI2 healthy mice (n=3) analysed at day 0 (T0) (m) and at day 30 after IRI (n=3, IRI T30) (n). Scale bars 40 µm.

o-q) Experimental scheme and representative images of the absence of basal Cre recombinase activity in the kidney of Pax2/FUCCI2 healthy mice (n=3) analysed at day 0 (T0) (p) and at day 30 after IRI (n=3, IRI T30) (q).

r) Percentage of p-H3+ cells over Phalloidin+ TECs at day 0 (T0), at day 2 after IRI (IRI T2) and at day 30 after IRI (IRI T30) (n=3 per group).

Scale bars 40 µm.

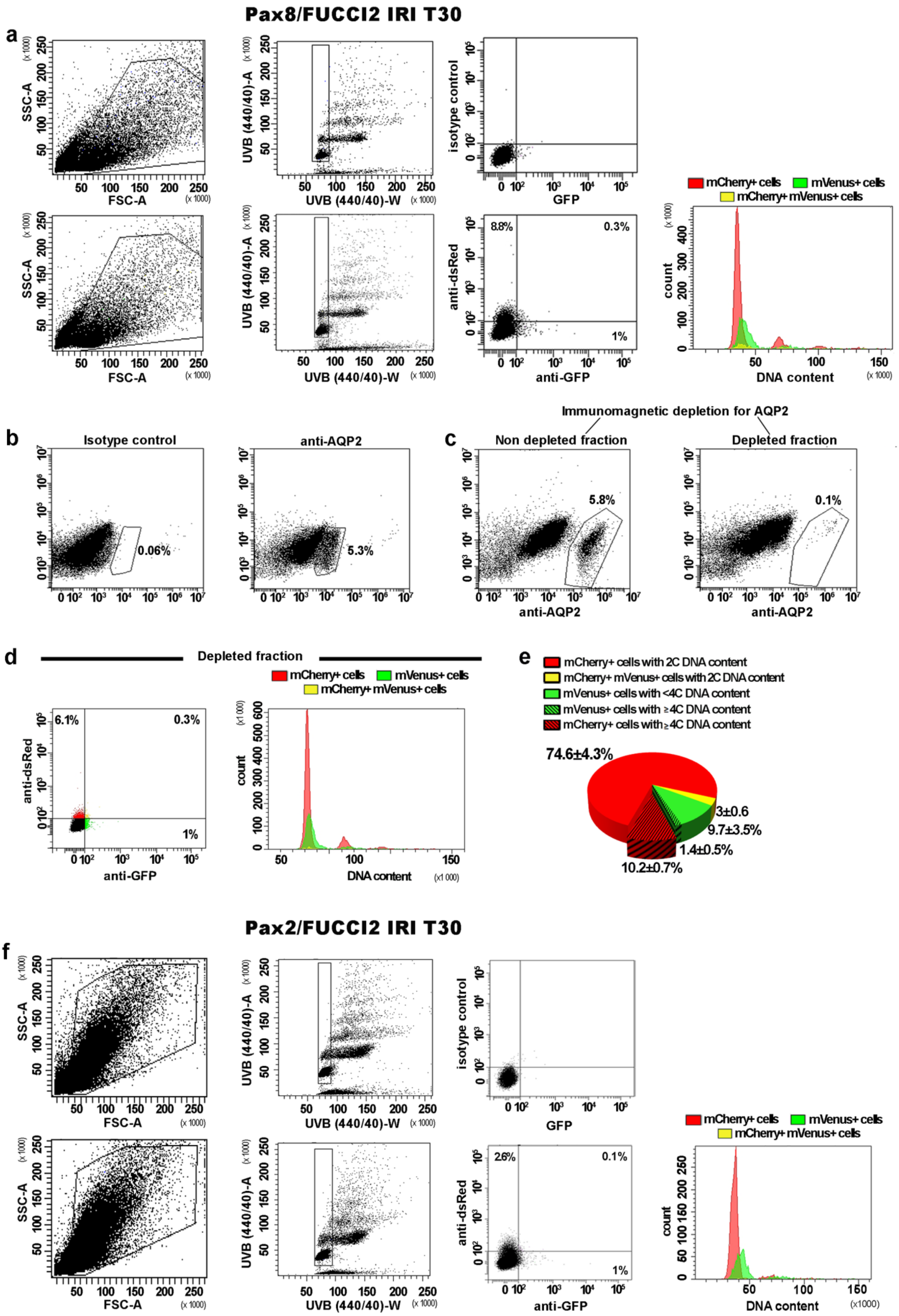
Pax8/FUCCI2 IRI T30
a
SSC-A
FSC-A
UVB (440/40)-A
UVB (440/40)-W
isotype control
GFP
8.8%
0.3%
1%
anti-dsRed
anti-GFP
mCherry+ cells
mVenus+ cells
mCherry+ mVenus+ cells
count
DNA content
b
Isotype control
anti-AQP2
0.06%
5.3%
Immunomagnetic depletion for AQP2
c
Non depleted fraction
Depleted fraction
5.8%
0.1%
anti-AQP2
d
Depleted fraction
6.1%
0.3%
1%
e
mCherry+ cells with 2C DNA content
mCherry+ mVenus+ cells with 2C DNA content
mVenus+ cells with <4C DNA content
mVenus+ cells with ≥4C DNA content
mCherry+ cells with ≥4C DNA content
74.6±4.3%
3±0.6
9.7±3.5%
1.4±0.5%
10.2±0.7%
Pax2/FUCCI2 IRI T30
f
2.6%
0.1%
1%

Supplementary Figure 7. Gating strategy and additional cell-cycle analysis in FUCCI2 mice.

a) Gating strategy of freshly isolated total renal cells of Pax8/FUCCI2 mice at day 30 after IRI (IRI T30). A representative experiment out of 5 is shown. Same experiment shown in Fig. 6w.

b) FACS analysis showed the percentage of AQP2+ cells in freshly isolated total renal cells of Pax8/FUCCI2 mice at day 30 after IRI (n=3) compared to the isotype control. At least 10,000 cells were analysed for each mouse. A representative experiment out of 3 is shown.

c) Immunomagnetic depletion of AQP2+ cells. At least 10,000 cells were analysed for each mouse (n=3). A representative experiment out of 3 is shown.

d) FACS and cell-cycle analysis in Pax8/FUCCI2 mice after depletion of AQP2+ cells at day 30 after IRI (n=3). More than 50,000 cells were analysed for each mouse. A representative experiment out of 3 is shown.

e) Percentages of mCherry+ cells, mVenus+ cells and mCherry+ mVenus+ cells over total FUCCI2 cells based on DNA content in Pax8/FUCCI2 mice after depletion of AQP2+ cells at day 30 after IRI. Data are mean ± SEM from 3 mice.

f) Gating strategy of freshly isolated total renal cells of Pax2/FUCCI2 mice at day 30 after IRI (IRI T30). A representative experiment out of 5 is shown. Same experiment shown in Fig. 6x.

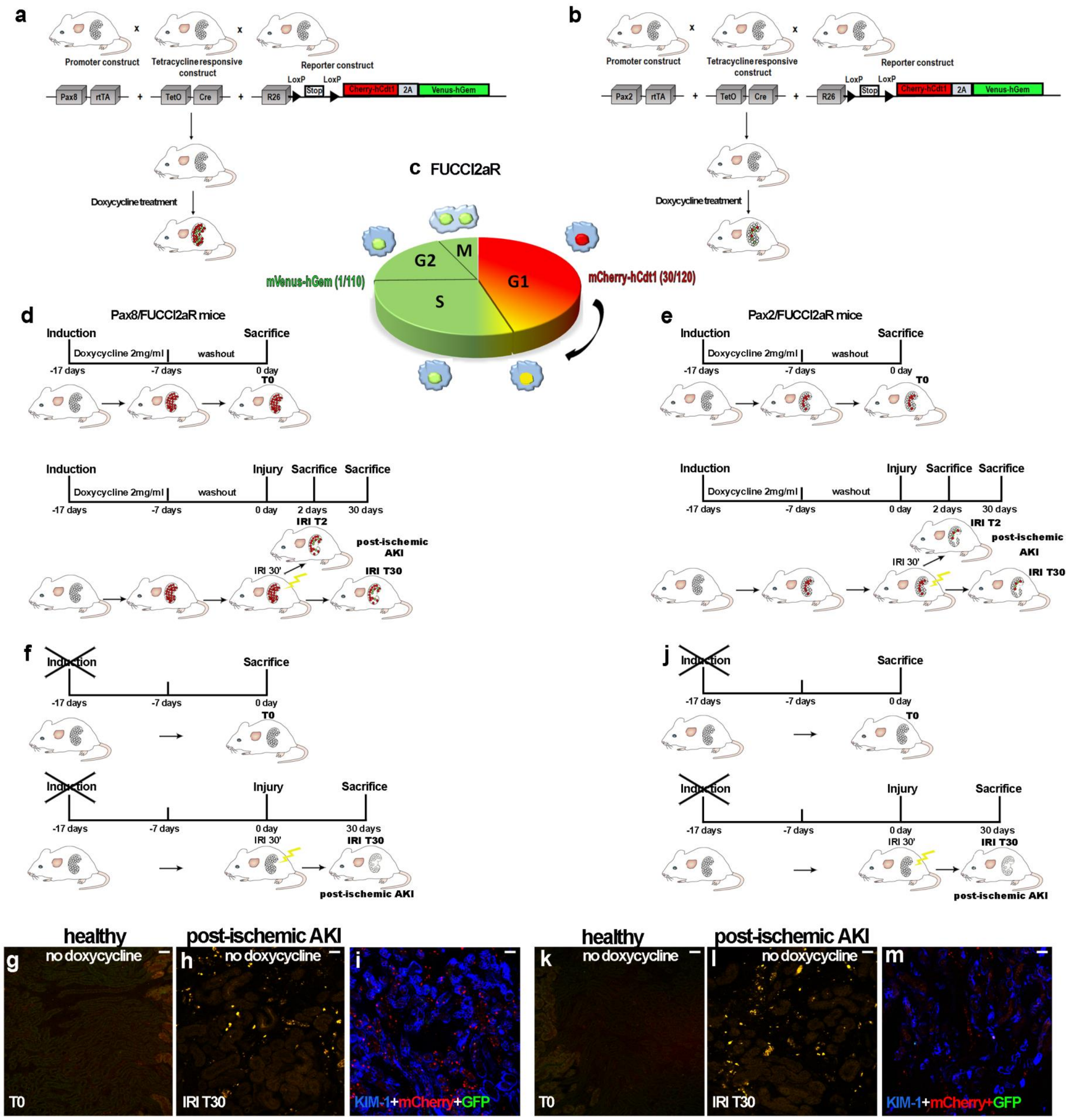

Supplementary Figure 8. Generation of inducible Pax8/FUCCI2aR mice and inducible Pax2/FUCCI2aR mice.

a) The inducible Pax8.rtTA;TetO.Cre;R26.FUCCI2aR (Pax8/FUCCI2aR) mouse is produced by crossing the Pax8.rtTA transgenic mice with TetO.Cre and Rosa26-FUCCI2aR transgenic mice. The triple transgenic mouse constitutively expresses rtTA in Pax8+ cells but does not express the reporter proteins in any cell type while maintained on water not containing doxycycline. When doxycycline is included in the water, Pax8+ cells express rtTA that binds the TetO element so that Cre recombinase expression is induced. The Cre

protein will specifically cut out the floxed Stop cassette and then turn on mCherry-hCdt1(30/120)-mVenus-hGem(1/110) expression. Even after withdrawal of doxycycline from the water, these Pax8+ cells will permanently express the reporters, whereas any new Pax8+ cells that develop after doxycycline exposure will not express the fluorescent reporters.

b) The inducible Pax2.rtTA;TetO.Cre;R26.FUCCI2aR (Pax2/FUCCI2aR) mouse is produced by crossing the Pax2.rtTA transgenic mice with TetO.Cre and Rosa26-FUCCI2aR transgenic mice. The triple transgenic mouse constitutively expresses rtTA in Pax2+ cells but does not express the reporter proteins in any cell type while maintained on water not containing doxycycline. When doxycycline is included in the water, Pax2+ cells express rtTA that binds the TetO element so that Cre recombinase expression is induced. The Cre protein will specifically cut out the floxed Stop cassette and then turn on mCherry-hCdt1(30/120)-mVenus-hGem (1/110) expression. Even after withdrawal of doxycycline from the water, these Pax2+ cells will permanently express the reporters, whereas any new Pax2+ cells that develop after doxycycline exposure will not express the fluorescent reporters.

c) Schematic representation of cell-cycle phases labelled with mCherry-hCdt1(30/120) and mVenus-hGem (1/110) reporters. mCherry-hCdt1 (30/120) is expressed in G1, whereas mVenus-hGem (1/110) is expressed in S/G2/M. Cells at the G1/S boundary express both mCherry-hCdt1(30/120) that mVenus-hGem (1/110). G1, gap 1 phase; S, synthesis; G2, gap 2 phase; M, mitosis.

d, e) Experimental schemes.

f-h) Experimental schemes and representative images of the absence of basal Cre recombinase activity in the kidney of Pax8/FUCCI2aR healthy mice (n=3) analysed at day 0 (T0) (g) and at day 30 after IRI (n=3, IRI T30) (h).

i) Expression of KIM-1(blue) in Pax8/FUCCI2aR at day 30 after IRI (n=5).

j-l) Experimental schemes and representative images of the absence of basal Cre recombinase activity in the kidney of Pax2/FUCCI2aR healthy mice (n=3) analysed at day 0 (T0) (k) and at day 30 after IRI (n=3, IRI T30) (l).

m) Expression of KIM-1 (blue) in Pax2/FUCCI2aR at day 30 after IRI (n=5).

Scale bars 40 µm.

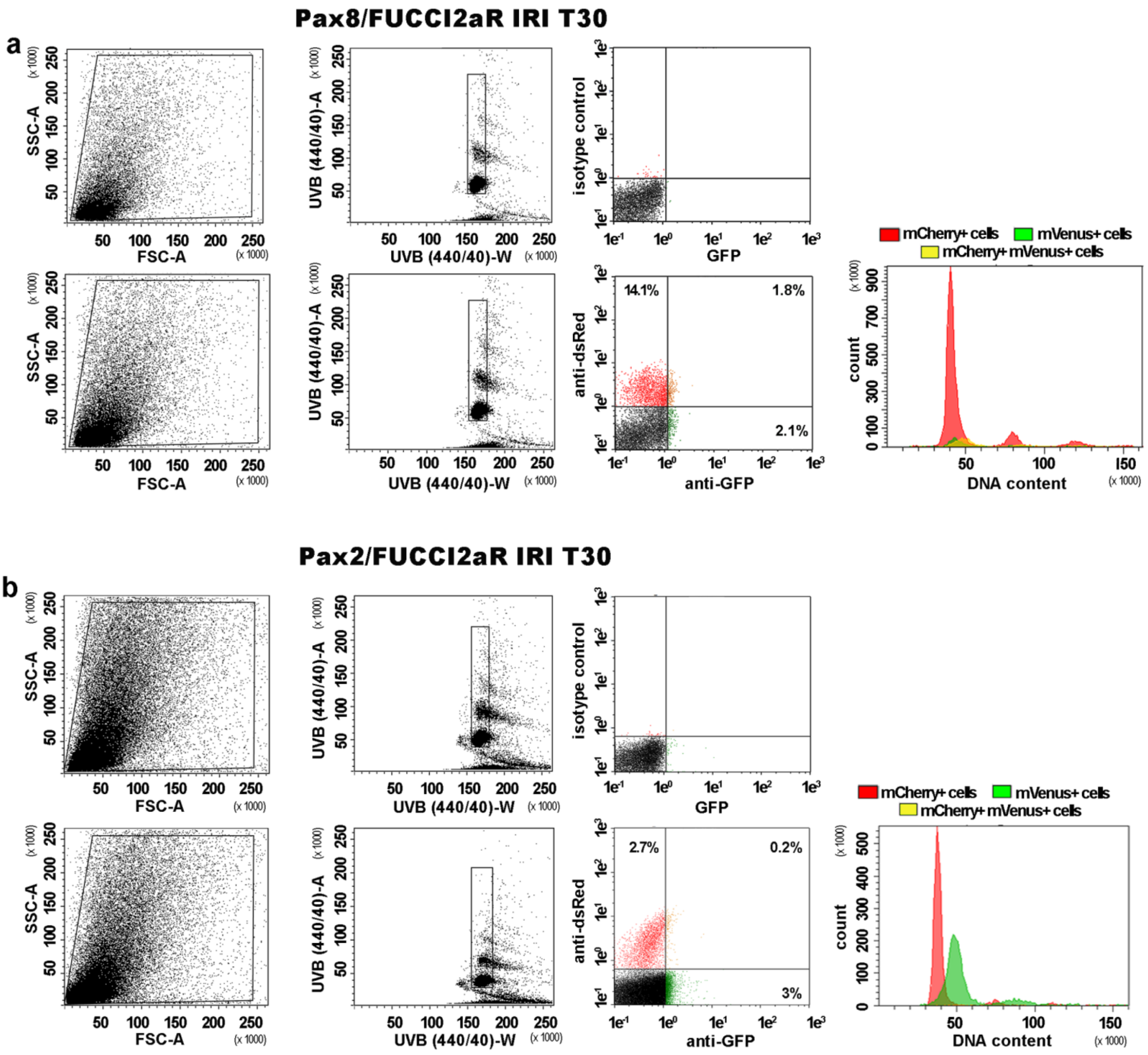

Supplementary Figure 9. Gating strategy in Pax8/ FUCCI2aR and Pax2/ FUCCI2aR mice

a) Gating strategy of freshly isolated total renal cells of Pax8/FUCCI2aR mice at day 30 after IRI (IRI T30) . A representative experiment out of 4 is shown. Same experiment shown in Fig. 7,h.

b) Gating strategy of freshly isolated total renal cells of Pax2/FUCCI2aR mice at day 30 after IRI (IRI T30). A representative experiment out of 4 is shown. Same experiment shown in Fig. 7i.

Supplementary Methods

Representative calculations in Pax2/Confetti mice

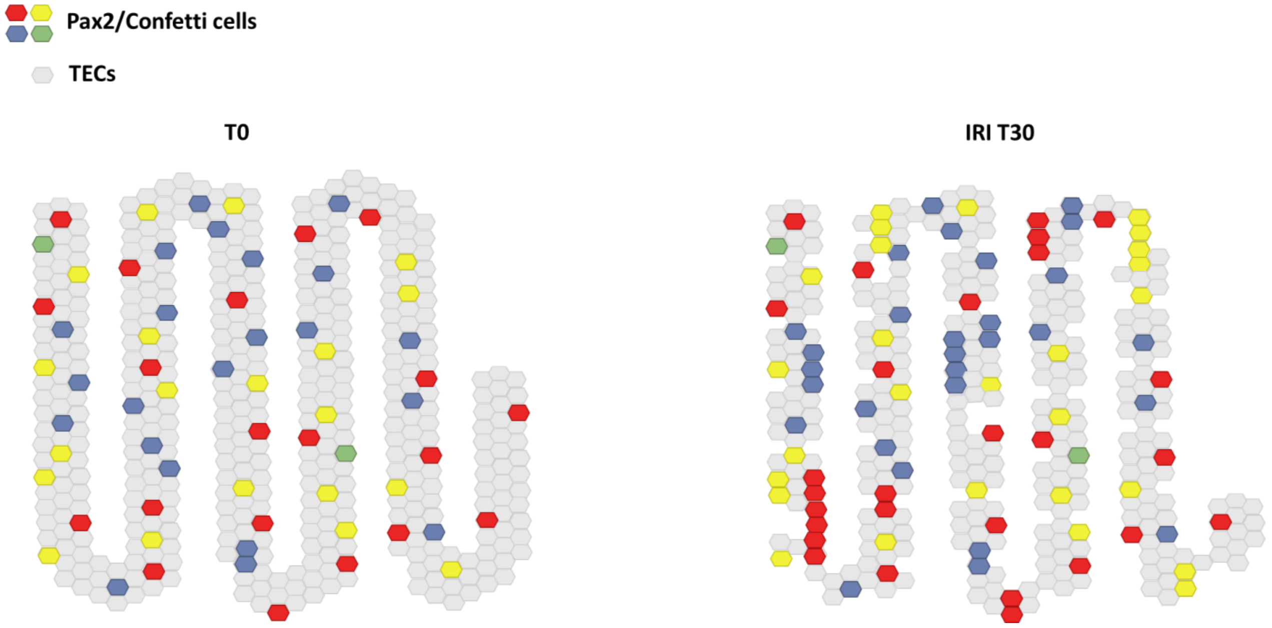

Clone size	n° of clones
1 cell	62
2 cells	1
3 cells	0
4 cells	0
5 cells	0
6 cells	0
**Total n° of clones**	63
**Total Pax2+ cells**	64

Clone size	n° of clones
1 cell	49
2 cells	7
3 cells	3
4 cells	2
5 cells	0
6 cells	1
**Total n° of clones**	62
**Total Pax2+ cells**	86

<u>Clone frequency of Pax2+ cells at IRI T30 (Equation I in Methods)</u>

$$(n° \; of \; clones \; of \; \mathbf{n} \; cells/total \; n° of \; clones) \times 100$$

Clone size	n° of clones	Clone frequency
1 cell	49	$49/62 \times 100 = \mathbf{79.03}\%$
2 cells	7	$7/62 \times 100 = \mathbf{11.29}\%$
3 cells	3	$3/62 \times 100 = \mathbf{4.83}\%$
4 cells	2	$2/62 \times 100 = \mathbf{3.22}\%$
5 cells	0	0
6 cells	1	$1/62 \times 100 = \mathbf{1.61}\%$

<u>Percentage of clonogenic cells (Equation II in Methods)</u>

A) n° of new clones at IRI T30 =

$$\sum_{\boldsymbol{n}=2}^{9}(clones\ composed\ of\ \boldsymbol{n}\ cells\ at\ IRI\ T30 - clones\ composed\ of\ \boldsymbol{n}\ cells\ at\ T0)$$

B) % of clonogenic cells at IRI T30 in comparison to T0

$$(A/\ n°\ of\ Pax2^{+} cells\ at\ T0)\ \times\ 100$$

<u>In this example</u>:

Clone size	n° at IRI T30	n° at T0	n° of new clones
2 cells	7	1	$7 - 1 = 6$
3 cells	3	0	$3 - 0 = 3$
4 cells	2	0	$2 - 0 = 2$
5 cells	0	0	0
6 cells	1	0	$1 - 0 = 1$

A) $\sum_{\boldsymbol{n}=2}^{6}(6; 3; 2; 0; 1 = 12)$

B) $(12/\ 64)\ \times\ 100 = \mathbf{18.75\%}$

<u>Percentage of new Pax2+ cells at IRI T30 (Equation III in Methods)</u>

C) n° of Pax2+ cells included in clones at IRI T30

$$\sum_{\boldsymbol{n}=2}^{9}[(clones\ composed\ of\ \boldsymbol{n}\ cells\ at\ IRI\ T30 - clones\ composed\ of\ \boldsymbol{n}\ cells\ at\ T0) \times \boldsymbol{n}]$$

D) n° of new Pax2+ cells at IRI T30= n° of Pax2+ cells included in new clones – n° of cells that originated new clones*

$$C - A$$

*the n° of cells that originated new clones coincides with the n° of new clones at IRI T30 (A), because each clone is the progeny of one cell

E) % of new Pax2+ cells at IRI T30 in comparison to T0

$$(D/\ n°\ of\ Pax2^{+} cells\ at\ T0) \times 100$$

In this example

Clone size	n° of new clones	n° of Pax2+ cells included in clones	n° of new Pax2+ cells
2 cells	6	$6 \times 2 = 12$	$12 - 6 = 6$
3 cells	3	$3 \times 3 = 9$	$9 - 3 = 6$
4 cells	2	$2 \times 4 = 8$	$8 - 2 = 6$
5 cells	0	0	0
6 cells	1	$1 \times 6 = 6$	$6 - 1 = 5$

C) $\sum_{\boldsymbol{n}=2}^{6}[12;9;8;0;6] = 35$

D) $35 - 12 = 23$

E) $(23/64) \times 100 = \mathbf{35.9}\%$

Percentage of lost Pax2+ cells at IRI T30 (Equation IV in Methods)

$$100 - [(n° \; of \; Pax2^{+} cells \; at \; IRI \; T30 - D / n° \; of \; Pax2^{+} cells \; at \; T0) \times 100]$$

In this example

$$100 - [(86 - 23)/64) \times 100] = \mathbf{1.56}\%$$
