# Supplementary material for: Endocycle-related tubular cell hypertrophy and progenitor proliferation recover renal function after acute kidney injury
Source: Nat Commun. 2018 Apr 9;9:1344. doi: 10.1038/s41467-018-03753-4 (PMC5890293; doi:10.1038/s41467-018-03753-4)
Supplement: Supplementary file 3 — Description of Additional Supplementary Files [file 41467_2018_3753_MOESM3_ESM.pdf]

**Description of Additional Supplementary Files**

File Name: Supplementary Movie 1
Description: 3D analysis of a Pax2+ clone regenerating a long tubule segment.
